# The effect and mechanism of exercise for post-stroke pain

**DOI:** 10.3389/fnmol.2022.1074205

**Published:** 2022-12-02

**Authors:** Yue Ma, Jing Luo, Xue-Qiang Wang

**Affiliations:** ^1^Department of Sport Rehabilitation, Xi’an Physical Education University, Xi’an, China; ^2^Department of Sport Rehabilitation, Shanghai University of Sport, Shanghai, China; ^3^Department of Rehabilitation Medicine, Shanghai Shangti Orthopaedic Hospital, Shanghai, China

**Keywords:** stroke, post-stroke pain, exercise, analgesic effect, analgesic mechanism

## Abstract

One of the common negative effects of a stroke that seriously lowers patients’ quality of life is post-stroke pain (PSP). Thus, exercise in PSP management has become a hot research topic. The main advantages of exercise therapy are affordability and ease of acceptance by patients compared to other treatment methods. Therefore, this article reviews the effectiveness and possible mechanisms of exercise interventions for PSP. Exercise training for patients with PSP not only improves physical function but also effectively reduces pain intensity and attenuates the behavioral response to pain. In addition, exercise therapy can improve brain function and modulate levels of pro-inflammatory and neurotrophic factors to exert specific analgesic effects. Potential mechanisms for exercise intervention include modulation of synaptic plasticity in the anterior cingulate gyrus, modulation of endogenous opioids *in vivo*, reversal of brain-derived neurotrophic factor overexpression, inhibition of purinergic receptor (P2X4R, P2X7R) expression, and inhibition of microglia activation. However, current research on exercise for PSP remains limited, and the sustainable benefits of exercise interventions for PSP need to be further investigated.

## Introduction

The health and lives of middle-aged and elderly adults are in jeopardy given that stroke is one of the primary causes of chronic impairment (Alawieh et al., 2018). Stroke also contributes to the high cost of treatment. However, a portion of that cost is used to treat secondary problems caused by strokes, such as pain, sensory impairment, cognition, memory, and balance problems (Han et al., 2017). Post-stroke pain (PSP) refers to a syndrome that is commonly associated with post-stroke complications caused by cerebrovascular accidents and corresponding vascular lesions. It mainly includes hemiplegic shoulder pain, central post-stroke pain, pain caused by spasticity, headache, and complex regional pain syndrome (Hansen et al., 2012). The main manifestations of PSP are ectopic pain, nociceptive hyperalgesia, and spontaneous pain. In addition, PSP can lead to anxiety and depression (Alagoz et al., 2018), thus affecting patients’ quality of life. More than half of the patients experienced pain 6 months after the stroke, and 33.6% had a moderate to heavy effect on activities of daily living because of pain (Hansen et al., 2012). Moreover, the pain has been associated with decreasing cognitive ability and increasing dependence on function. However, PSP can often be ignored or not adequately treated by the patient (Harrison and Field, 2015; Paolucci et al., 2016).

The current treatment for PSP includes pharmacological and non-pharmacological therapies. Although the primary treatment for PSP is pharmacological, non-pharmacological approaches have increased in recent years. In particular, the application of exercise interventions in PSP management has received much attention. Compared to other treatments, exercise therapy is affordable and easy to perform, which makes it acceptable to patients. Previous studies have demonstrated that exercise could reduce pain sensitivity and increase pain thresholds (Jones et al., 2014; Belavy et al., 2021; Peng et al., 2022; Wu et al., 2022). For stroke patients, exercise can improve trunk stability, enhance balance and walking ability to reduce the incidence of falls (Lee and Stone, 2020; Jung et al., 2021). It has been shown that exercise can improve cognitive function and activities of daily living in stroke patients (Li et al., 2022; Nindorera et al., 2022). Physical exercise is also effective in improving patients’ quality of life and depressive symptoms after stroke (Ali et al., 2021; Zhang W. et al., 2021). However, there is still a lack of research on the mechanisms of exercise interventions for PSP. Therefore, this review discusses the clinical efficacy and possible mechanisms of exercise on PSP.

## Effect of exercise on PSP

Exercise intervention is a simple, cost-effective, and widely applicable treatment for stroke patients. Meanwhile, exercise can improve cardiovascular health and physical function to avoid subsequent strokes (Saunders et al., 2014). Exercise guidelines for stroke also recommend 3–5 days of aerobic activity or 2–3 days of resistance exercise per week for adults with mild to moderate stroke (Kim Y. et al., 2019). However, PSP remains a complex medical problem to solve. It has been reported that the onset of PSP is associated with activity restriction (Atalan et al., 2021). Thus, the presence of pain hinders the rehabilitation process and affects the patient’s daily life. Various types of exercise are effective in relieving stroke pain, including strength training, aerobic exercise, stretching, and flexibility training, among others (Zhang Y. H. et al., 2021). Strength training can improve muscle weakness, increase trunk and lower extremity stability, improve walking ability, and improve overall function and quality of life in stroke patients (Han et al., 2017). Strength training has also been found to have a positive effect on reducing pain by increasing pain thresholds and decreasing pain sensitivity (Assa et al., 2019). The Monkey Chair and Band Exercise System Training for Stroke Patients was a randomized controlled trial performed by Jeon et al. (2016). This exercise system included joint motion, strengthening training, and relaxation. Meanwhile, using the visual analog scale (VAS), they found a progressive and significant improvement in VAS scores over time in the experimental group trained with the exercise intervention comparing the controls. This discovery indicates that exercise interventions are effective in reducing PSP. Aerobic exercise is a major component of stroke rehabilitation and cardiac rehabilitation and is a valuable intervention to promote cardiovascular health in stroke patients. Aerobic exercise can exert analgesic effects by inducing hyperalgesia, reducing musculoskeletal pain, and decreasing pain sensitivity in both healthy and chronic pain populations (Öte Karaca et al., 2017; Wewege and Jones, 2021). Complex regional syndrome (CPRS) is one of the common types of post-stroke pain. It is a diffuse pain, usually with swelling and vasodilatation changes in the limb, which severely affects the patient’s physical movement (Delpont et al., 2018). Topcuoglu et al. (2015) recruited a total of 40 post-stroke CRPS type I patients who were randomized to a training group for the aerobic exercise of the upper extremities and a control group for conventional physical therapy for a 4-week intervention while using the VAS for pain assessment. They found that the training group of patients had significantly lower pain and fewer signs and symptoms of CRPS at the end of treatment. Flexibility training can reduce the pain of musculoskeletal problems from stroke by easing muscle spasms, improving muscle tone, and reducing joint contractures through slow stretching. As one of the flexibility exercises, yoga training can effectively improve fine motor, balance, flexibility, and quality of life in stroke patients (Bastille and Gill-Body, 2004; Lynton et al., 2007; Immink et al., 2014). In addition, yoga training has been shown to be effective in relieving post-stroke pain. Schmid et al. (2014) recruited a total of 47 chronic stroke patients for 8 weeks of therapeutic yoga training. Herein, a PEG was used to assess pain intensity, which is a 3-item physical functional measurement of pain. They discovered that 8 weeks of therapeutic yoga training markedly reduced the intensity of PSP. Thus, this finding suggests that exercise interventions can help treat pain after a stroke. Table 1 provides more details of studies on exercise interventions for PSP.

**Table 1:** General characteristics of studies on exercise for PSP.

Study	Participants			Exercise intervention		Pain outcomes	Result
	Population	Sample size	Mean age(years)	Exercise type	Exercise duration		
Costantino et al. (2017)	Patients with chronic post-stroke	N = 32 E: n = 17 C: n = 15	61.59 E:62.59C:60.47	Local muscle vibration during voluntary isometric contraction	30 min 3 times per week, 12 sessions	VNRS	Significant improvement in pain
Jeon et al. (2016)	Patients with post-stroke Hemiparesis	N = 12 E: n = 6 C: n = 6	E:58.0C:50.5	The Monkey Chair and Band exercise	30 min per session; 3 times per week;12 weeks	VAS	The significant difference in pain VAS scores
Wei et al. (2019)	Patients with hemiplegic shoulder pain	N = 40 E: n = 20 C: n = 20	E:63.85C:65.55	Acupuncture combined with neuromuscular joint facilitation	Once a day and 6 times per week over 3 weeks	VAS	Significant improvement in VAS scores
Kim M. S. et al. (2019)	Patients with Hemiplegic shoulder pain	N = 36 E: n = 18 C: n = 18	65.3 E:65.9C:64.7	Robotic-assisted joint mobilization and stretching exercises	30 min per day, 5 times per week for 4 weeks	VAS	VAS scores improved more in the intervention group than control group
Lee and Kim (2013)	Patients with stroke	N = 7	68.1	Self-directed exercise with the task board	Once a week for 10 weeks	VAS	The scores of the VAS were improved
Schmid et al. (2014)	Patients with Chronic stroke	N = 47 E:37 C:10	63.1 E:63.9C:60.2	Group therapeutic-yoga	1 h per time, twice a week for 8 weeks	PEG	Pain scores significantly improved after 8 weeks of yoga
Topcuoglu et al. (2015)	Patients with CRPS I after stroke	N = 40 E: n = 20 C: n = 20	E:65.95C:67.5	Upper-extremity aerobic exercise	30 min5 times per week over 4 weeks	VPS	Pain reduction and significant improvement in CRPS signs and symptoms

C, control group; CPRS I, complex regional pain syndrome type I; E, exercise group; PEG, P, mean pain intensity, E, disturbance to enjoy life, G, disturbance to general practice; VAS, Visual Analog Scale; VNRS, Verbal Numerical Rating Scale of pain; VPS, Visual Pain Scale.

## Mechanisms of exercise for PSP

**Figure 1:**
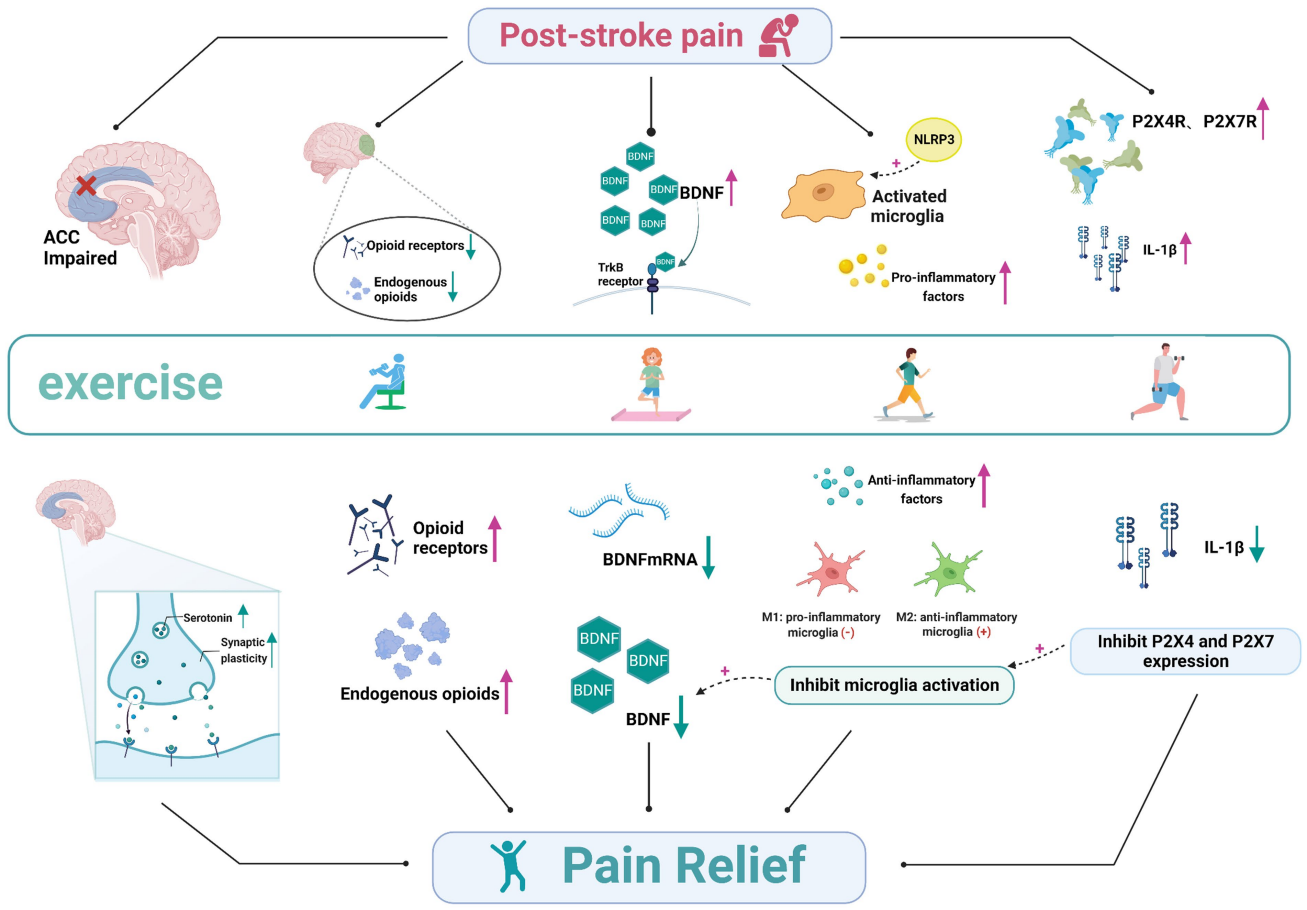
The involved mechanisms in exercise on post-stroke pain. The analgesic mechanism of exercise for post-stroke pain, involving the regulation of the synaptic plasticity in the anterior cingulate cortex regulation of endogenous opioids *in vivo*, reversal of brain-derived neurotrophic factor overexpression, inhibition of purinergic receptor (P2X4R, P2X7R) expression, and inhibition of microglia activation. ACC, anterior cingulate cortex; TrkB, tyrosine receptor kinase B; NLRP3, the Nod-like receptor family pyrin domain containing 3 (NLRP3); IL-1β, interleukin-1β.

Exercise can enhance brain function and reduce the degeneration of nerves. Exercise can also alter post-stroke neural networks, neuronal excitability, and neurotrophic factors, thereby affecting neuroplasticity (Penna et al., 2021). Meanwhile, activity can modulate the levels of pro-inflammatory cytokines, reduce nociceptive hypersensitivity, and attenuate behavioral pain responses to exert specific analgesic effects. PSP is associated with both neurological and nociceptive mechanisms. There are several possible mechanisms by which exercise interventions improve PSP, including the regulation of the synaptic plasticity in the anterior cingulate cortex (ACC), regulation of endogenous opioids *in vivo*, reversal of brain-derived neurotrophic factor (BDNF) overexpression, inhibition of purinergic receptor (P2X4R, P2X7R) expression, and inhibition of microglia activation (Figure 1).

### The regulation of synaptic plasticity in ACC

Numerous studies have shown that the spinal thalamic tract and thalamic cingulate pathway are engaged in the production of PSP, whose damage is caused by stroke pain (Ning and Oaklander, 2008; Sprenger et al., 2012; Lu et al., 2015). The pain information from the soma and viscera is delivered to the anterior cingulate cortex (ACC) primarily through three projection systems (thalamus, amygdala, and other pain-related cortical areas). Long-term inhibition (LTD) and Long-term potentiation (LTP) are formed for synaptic plasticity. LTD and LTP are causally related to chronic pain at the dorsal horn of the spinal cord and cortex regions, including ACC (Bliss et al., 2016). Remarkably, the ACC is an essential portion of the pain pathway and is a critical brain region for processing pain information. Seghier et al. found that the ACC and parietal regions had pain-specific signals altered at different thermal sensory stimuli by functional MRI, thereby suggesting that the ACC is involved in mediating the pain response in PSP patients (Seghier et al., 2005). Various previous studies have confirmed the regulation of ACC by exercise. Meanwhile, Zhou et al. (2022) explored the influence on pain relief of regular aerobic training by recording changes in neuronal activity and synaptic plasticity of ACC using a voluntary running wheel. Thus, the results showed that regular exercise enhanced the release of serotonin and regulated ACC synaptic plasticity, which reduced pain intensity through 5-HT1A and 5-HT7 receptor functions (serotonin may be a pain modulator). Herein, we might consequently surmise that exercise reduces PSP by modulating the ACC’s synaptic plasticity.

### The modulation of endogenous opioids

Endogenous opioids are expressed throughout the central and peripheral nervous system while regulating various neuronal pathways and functions, one of the essential functions being the modulation of the pain response. Central stroke pain caused by cerebral infarction is strongly associated with changes in specific central structures and conduction pathways of opioids. Opioid neurogenic mechanisms are involved in the neurotransmission of central pathological pain (Willoch et al., 1999, 2004). Willoch et al. performed a study using the non-selective ligand [11C] diprenorphine (DPN), PET, to measure altered opioid receptor incorporation for patients experiencing PSP while comparing with controls (Willoch et al., 2004). Herein, their findings suggest that central structures associated with pain (including ACC and lateral prefrontal cortex) indicate reduced opioid receptor binding. The periaqueductal gray (PAG) and rostral medial medulla (RVM) are important components of the downstream regulatory pathways of pain. Meanwhile, Nicola et al. carried out research where 5 weeks of running wheel exercise increased endogenous opioid concentrations in mice PAG and RVM, which further suggests that exercise regularly increases endogenous opioid expression and reverses neuropathic pain mediated through the central nervous system (Stagg et al., 2011). In addition, de Oliveira et al. observed that 45 days of long-and short-term training increased the levels of MOR opioid receptors in rat hippocampus structures (de Oliveira et al., 2010). Furthermore, Koltyn et al. showed that exercise produces analgesia primarily by mediating endogenous opioids (Koltyn, 2000). Hence, exercise can increase endogenous opioid levels (Hoffmann et al., 1990; Debruille et al., 1999). Meanwhile, in a survey by Mazzardo-Martins et al., prolonged high-intensity swimming training diminished the pain response in experimental rats through a combination of mechanisms, including activation of opioids and increased release of endogenous opioids from the adrenal glands (Mazzardo-Martins et al., 2010). Thus, exercise can have an analgesic effect by modulating endogenous opioids in the cerebral cortex and associated pain-related transmission pathways.

### The modulation of BDNF levels

Brain-derived neurotrophic factor (BDNF) possesses the potential to promote brain plasticity and is a key player in exercise-induced neuroprotection following ischemic stroke, which has become a key facilitator of neuroplasticity in post-stroke recovery (Mang et al., 2013). In addition, BDNF is associated with cognition and memory but can be involved in transmitting pain. The dorsal root ganglion (DRG) produces BDNF, which is then carried to primary sensory afferent centers for release into the dorsal horn of the spinal cord. Whereby, it connects to tyrosine receptor kinase B (TrkB) receptors in the secondary sensory neurons and performs synaptic transmission and nociceptive neuromodulation (Obata and Noguchi, 2006). Following central nerve injury, BDNF release stimulates nociceptive receptors, thereby developing nociceptive hypersensitivity. In pathological states such as CPSP, elevated BDNF levels lead to over-binding with TrkB receptors, thus leading to a high frequency of neuronal bursts along the thalamic cingulate pathway (Kuan et al., 2018).

Mariacristina et al. examined the levels of BDNF in 50 stroke patients and discovered that those with subacute stroke pain had significantly higher levels of BDNF and a considerable overexpression of BDNF (Siotto et al., 2017). It has been shown that exercise could affect the expression of BDNF (Berchtold et al., 2005). Exercise training could reduce the overexpression of BDNF in DRG. Moreover, numerous studies have demonstrated that exercise therapies boost the normalization of neurotrophic growth factors in various diseases, including stroke, cerebrospinal cord injury, major depression, and anxiety disorders (Hutchinson et al., 2004; Ang and Gomez-Pinilla, 2007). Almeida et al. also found that treadmill exercise reduced the expression of BDNF mRNA after neurological damage and promoted neurological recovery; plate exercise reduced hypoxia-induced activation of hippocampal astrocytes and microglia in rats, thereby restoring BDNF levels (Almeida et al., 2015). Therefore, we can conclude that exercise reverses BDNF overexpression, thus resulting in the alleviation of PSP.

### The inhibition of purinergic receptors expressions

Numerous earlier investigations have demonstrated the connection between pain and purinergic receptors, which are implicated in the transmission of pain. Burnstock has also proposed a unified purinergic hypothesis for pain initiation (Burnstock, 1996). Purinoceptors are a signaling system prevalent in the human body. Thus, sensory nerve endings can express purinergic receptors in many circumstances. P2X4 and P2X7 receptors have essential modulatory roles in neuropathic pain, and their antagonists may reduce pain. One promising approach to pain treatment could be the modulation of purinergic receptors. Lu et al. found a significant increase in the expression of microglia P2X4 receptors in the tissue surrounding thalamic lesions after cerebral hemorrhage, and mechanical pain in central post-stroke pain rats was reversed by blocking P2X4 receptors (Lu et al., 2021a). Meanwhile, Shih et al. used an intra-thalamic injection of collagenase to induce central stroke pain and found increased expression of P2X4 receptors in mice. This result demonstrates that purinergic P2X4 receptors are associated with stroke pain (Shih et al., 2017). In addition, the activated P2X4R is associated with BDNF release from microglia, which can modulate stroke pain by regulating BDNF.

A common pain phenotype is shared by P2X4 and P2X7 receptors in knockout mice. P2X7 receptors have a strong relationship with the pro-inflammatory cytokine IL-1, which is principally engaged in the transmission of pain and was released. However, low P2X7R expression does not harm the nervous system under normal circumstances, but when P2X7R is activated by pathological situations such as cerebral ischemia, tissue damage increases. Simultaneously, P2X7 receptor overexpression can also cause tissue damage. Zhang et al. discovered that P2X7R was substantially expressed in neurons following middle cerebral artery blockage. Furthermore, P2X7R overexpression stimulated microglia, increased cell membrane permeability, generated proinflammatory factors, and even further exacerbated neuronal damage (Inoue and Tsuda, 2021). There is growing evidence that P2X7 receptors play a particular function in persistent pain. According to research by Chessell et al., inhibiting P2X7 receptors decreased pain perception across animal studies of both acute and persistent neuropathic pain (Chessell et al., 2005). P2X7R and interleukin-1β (IL-1β) expression levels were discovered to be increased in microglia inside the spinal cord’s dorsal horn cord by Zhou et al. in an animal study of pain. In addition, P2X7R antagonists were used to drastically diminish P2X7R and IL-1β expression in its spinal cord and relieve pain (Zhou et al., 2019). This further suggests that selective blockade of P2X7 receptor expression in the body may reduce pain. Thus, we speculated that inhibiting P2X4 and P2X7 receptor expression could alleviate PSP.

Herein, the role of exercise in inhibiting the expression of purinergic receptors (P2X4R, P2X7R) has been studied. Exercise reduces the progression of pain by activating or inhibiting the expression of BDNF, which is achieved by changing extracellular nucleotide profiles along with purinoceptors in the structures of the central nervous system, particularly P2X4 and P2X7 receptors (Sun et al., 2022). Grace et al. conducted research and found that the expression of neuroexcitatory interleukin-1β (IL-1β) in the ipsilateral dorsal horn of the spinal cord was normalized and glutamate transporter (GLT-1) was reduced by voluntary wheeling, whereas the expression of P2X4 receptors was inhibited (Grace et al., 2016). Meanwhile, a study conducted by Chen et al. showed that the upregulation of P2X7 receptor levels was reversed in mice in the exercise group as compared with controls by 12 weeks of treadmill training (Chen et al., 2019). Therefore, we may conclude that exercise produces analgesia for PSP by modulating the expression of purinergic receptors (P2X4R, P2X7R).

### Inhibition of microglia activation

Immune cells called microglia are found in the central nervous system. They participate in the development of the nervous system’s angiogenesis, apoptosis induction, phagocytic removal of dead cells, and synaptic remodeling (Eyo and Dailey, 2013). PSP production is also closely linked to microglia activation. Neurological damage activates microglia, thereby leading to enhanced production of pro-inflammatory factors, which cause painful symptoms (He et al., 2021). Damaged neurons and glia may also activate purinergic receptors (P2X4R, P2X7R) in microglia by increasing the release of purines (Eyo and Dailey, 2013). As mentioned above, these purinergic receptors are associated with PSP. It was found that microglia are involved in PSP development. Nagasaka et al. found that microglia and astrocytes around the lesion were activated after 3 months by establishing a stroke pain model in macaques (Nagasaka et al., 2017). In addition, the Nod-like receptor family pyrin domain containing 3 (NLRP3) inflammatory vesicles is associated with neurological diseases such as ischemic stroke. Li et al. summarize numerous previous studies of NLRP3 inflammatory vesicles and PSP, one of the mechanisms is that NLRP3 inflammatory vesicles cause thalamic lesions and reinforce microglia to undergo an inflammatory response. Prolonged inflammation inhibits ventral basal neurons’ function, thereby leading to central stroke pain (Li et al., 2018). Meanwhile, cerebral hemorrhage activates NLRP3 inflammatory vesicles and inflammation, microglia activation mediates the inflammatory response in the brain, and NLRP3 inflammatory vesicles amplify the inflammatory response. Thus, activation through microglia and NLRP3 inflammatory vesicles can eventually lead to PSP.

Exercise is an effective stroke treatment. In addition, exercise can inhibit the activation of microglia through the upregulation of anti-inflammatory cytokine expression (Mee-Inta et al., 2019). Furthermore, Lu et al. found that treadmill exercise increased interleukin 4 (IL-4) expression, decreased markers of pro-inflammatory M1 cells in microglia, and increased characteristics of anti-inflammatory M2 cells to inhibit M1 microglia and promoted M2 microglia activation, thereby suppressing the inflammatory response during the stroke (Lu et al., 2021b). Tamakoshi et al. found that performing ultra-early exercise after cerebral hemorrhage inhibited microglia activation by establishing a rat model of cerebral hemorrhage (Tamakoshi et al., 2022). Meanwhile, Almeida et al. discovered that extending the duration of swimming activity turned back the overactivity of astrocytes and microglia in the post-dorsal horn following nerve injury, thereby resulting in a reduction in pain behavior in mice (Almeida et al., 2015). Other studies have reported the inhibitory effect of exercise on microglia activation (He et al., 2017). Accordingly, we can conclude that exercise can relieve post-stroke pain by inhibiting microglia activation after cerebrovascular accidents.

## Conclusion

Most stroke patients suffer from pain, which severely affects the patient’s activities and daily life. Therefore, it is crucial to manage pain in post-stroke patients. Various exercises effectively improve PSP, including strength training, aerobic exercise, and yoga exercises. The alleviating effect of exercise on stroke pain can be achieved through enhanced ACC function of the brain cortex, modulation of endogenous opioids, brain-derived neurotrophic factors, purinergic receptor expression, and microglia activation. Therefore, this article reviews the potential mechanisms of exercise for PSP relief. Hence, it demonstrates the effectiveness and role of exercise interventions in PSP treatment and comfort, which may hopefully be useful for future applications of exercise therapy in stroke pain. Meanwhile, more attention should be paid to the persistent effect of exercise on PSP and the effect of different exercise parameters on pain relief in future studies.

## Author contributions

X-QW conceived this review. YM and JL wrote the first draft of the manuscript. YM, JL, and X-QW searched for relevant studies, then analyzed and organized the data. X-QW revised the form of the manuscript. All authors contributed to the article and approved the submitted version.

## Funding

This work was supported by the Shanghai Frontiers Science Research Base of Exercise and Metabolic Health, the Shanghai Key Lab of Human Performance (Shanghai University of Sport) (11DZ2261100), Talent Development Fund of Shanghai Municipal (2021081), and Shanghai Clinical Research Center for Rehabilitation Medicine (21MC1930200).

## Conflict of interest

The authors declare that the research was conducted in the absence of any commercial or financial relationships that could be construed as a potential conflict of interest.

## Publisher’s note

All claims expressed in this article are solely those of the authors and do not necessarily represent those of their affiliated organizations, or those of the publisher, the editors and the reviewers. Any product that may be evaluated in this article, or claim that may be made by its manufacturer, is not guaranteed or endorsed by the publisher.
